# Chronic Multi-Electrode Electromyography in Snakes

**DOI:** 10.3389/fnbeh.2021.761891

**Published:** 2022-01-07

**Authors:** Grady W. Jensen, Patrick van der Smagt, Harald Luksch, Hans Straka, Tobias Kohl

**Affiliations:** ^1^Graduate School of Systemic Neurosciences (GSN-LMU), Ludwig-Maximilians-University, Munich, Germany; ^2^ARGMAX.AI Volkswagen Group Machine Learning Research Lab, Munich, Germany; ^3^Department of Artificial Intelligence, Faculty of Informatics, Eötvös Lórand University, Budapest, Germany; ^4^Chair of Zoology, Technical University of Munich, Freising, Germany; ^5^Department Biology II, Ludwig-Maximilians-University of Munich, Munich, Germany

**Keywords:** EMG, electromyography, locomotion, reptile, snake, strike

## Abstract

Knowledge about body motion kinematics and underlying muscle contraction dynamics usually derives from electromyographic (EMG) recordings. However, acquisition of such signals in snakes is challenging because electrodes either attached to or implanted beneath the skin may unintentionally be removed by force or friction caused from undulatory motion, thus severely impeding chronic EMG recordings. Here, we present a reliable method for stable subdermal implantation of up to eight bipolar electrodes above the target muscles. The mechanical stability of the inserted electrodes and the overnight coverage of the snake body with a “sleeping bag” ensured the recording of reliable and robust chronic EMG activity. The utility of the technique was verified by daily acquisition of high signal-to-noise activity from all target sites over four consecutive days during stimulus-evoked postural reactions in Amazon tree boas and Western diamondback rattlesnakes. The successful demonstration of the chronic recording suggests that this technique can improve acute experiments by enabling the collection of larger data sets from single individuals.

## Introduction

Acquisition of long-term chronic EMG data is well-established in many animal species ranging from fish (Cooke et al., 2004), to mammals such as mouse (Tysseling et al., 2013), monkeys (Park et al., 2000), and even humans (Kern et al., 2001). However, electromyography (EMG) in reptiles employing invasive intramuscular electrode implantation is often limited to acute experiments extending over a few hours or a single day. Alternatively, multi-unit activity from muscle fibers can be recorded over a wider area with non-invasive surface electrodes (sEMG; Staudenmann et al., 2010), although with variable success in reptiles, given their generally rather solid, scaled skin. Thus, sEMG recordings in snakes are technically challenging because the signal is lost when the animals remove electrodes and/or recording devices while rubbing their body against objects. Since this affects the reliability of chronic data acquisition, sEMGs are rarely used for muscle activity recording in reptiles. Instead, bipolar hook wire electrodes are mostly implanted directly into the muscle tissue. In this case, electrode fixation is achieved through the barbed hook of the electrodes. Cyanoacrylate glue, vet-wrap adhesive bandage, and plastic cement is mostly used to additionally secure the electrode wires on the outside skin and to tie the wires to each other to form a single strand of wires (for a detailed description of this classic technique see: Jayne, 1988; Sharpe et al., 2013). This technique of intramuscular EMG recordings has been used previously to shed light on a variety of snake behaviors from locomotion (Jayne, 1988; Newman and Jayne, 2018) to feeding and drinking (Cundall and Ganz, 1979; Cundall, 1983; Berkhoudt et al., 1994) but also on more specific behaviors such as strikes (Young, 2010) or venom spitting (Young et al., 2004, 2009). There has even been a study on how epaxial muscles of the snake are activated during reaching tasks (Jorgensen and Jayne, 2017). In all these studies, 1–12 electrodes were inserted into locally restricted body segments either percutaneously or by surgically opening the skin to expose the muscles of interest. In general, animals were sacrificed after finishing EMG recordings, which was mostly on the same day as the electrode implantation, classified this approach as an acute experiment. However, Moon (2000) was successful in keeping bipolar hook electrodes in place within snake axial muscles for up to 1 week performing chronic EMG recordings.

Instead of using barbed bipolar electrodes, surface patch electrodes have been used in studies on salamanders (Carrier, 1993), iguanas (Carrier, 1989), and snakes (Moon and Gans, 1998). This type of electrodes facilitates the recording of EMG signals from muscles that are too thin to accommodate barbed bipolar electrodes (Carrier, 1989) or when muscles are in close proximity to adjacent, larger muscles that potentially generate cross-talk of the activity pattern (Loeb and Gans, 1986). In order to solve this issue, a patch, made of silicone rubber sheet was used to provide electrical insulation from the larger muscles, while electrical activity from the surface of the small muscle was recorded by the bipolar electrodes attached to the other side of the patch.

The main drawback of classic sEMG recordings, is the limited spatial precision, because this technique precludes a precise identification of the recorded muscle or the exact vertebral location during the actual experiments. However, in studies, such as our present investigation, in which spatio-temporal muscle activation patterns of larger body segments (e.g., loops) with respect to relative body position (inside vs. outside, rostral vs. caudal) or type of muscle activation (tonic vs. phasic) is the major goal, a high spatial resolution would be desirable but is not essential to obtain the respective information. To overcome the technical limitations of sEMG recordings in snakes and to allow for chronic multi-site muscle activity analyses, we have developed a new technique for collecting multi-channel EMG data from multiple body segments during the execution of a natural snake behavior. Our technique resembles the use of patch electrodes, with the skin providing electric insulation from one side, while electric activity is recorded from the surface of large epaxial muscles with bipolar electrodes. This recording technique avoids surgical exposure of the muscle or the use of barbed electrodes and is thus less invasive. This technique permits stable EMG recordings with a high signal-to-noise ratio. Both, individual spikes and local muscle group dynamics, normally associated with sEMG data, were robustly and consistently acquired in two species of snakes. Furthermore, this technique is suitable for the recording of high-quality data over several days and can thus be employed for chronic recording of EMG data in snakes.

## Materials and Methods

### Experimental Animals

In this study, five semi-adults [snout-vent length (SVL), range: 85–106 cm; body weight range: 79–174 g] Amazon tree boas (*Corallus hortulanus*) and two semi-adult Western diamondback rattlesnakes (*Crotalus atrox*, Baird and Girard, 1853) (SVL, range: 42–45 cm; body weight range: 112–122 g) were used. In addition, four carcasses of Amazon tree boas were used to establish the electrode implantation prior to the actual animal experiments. Snakes were bred at the Chair of Zoology (Technical University of Munich) and maintained on a 12 h:12 h light:dark regime, 22–33°C temperature range, and a diet of pre-killed rodents with water *ad libitum*. Permission for the experiments was granted by the respective governmental institution for animal welfare (Regierung von Oberbayern, Gz.: ROB-55.1-2532.Vet_02-19-115).

### Presurgical Preparation

**Figure 1:**
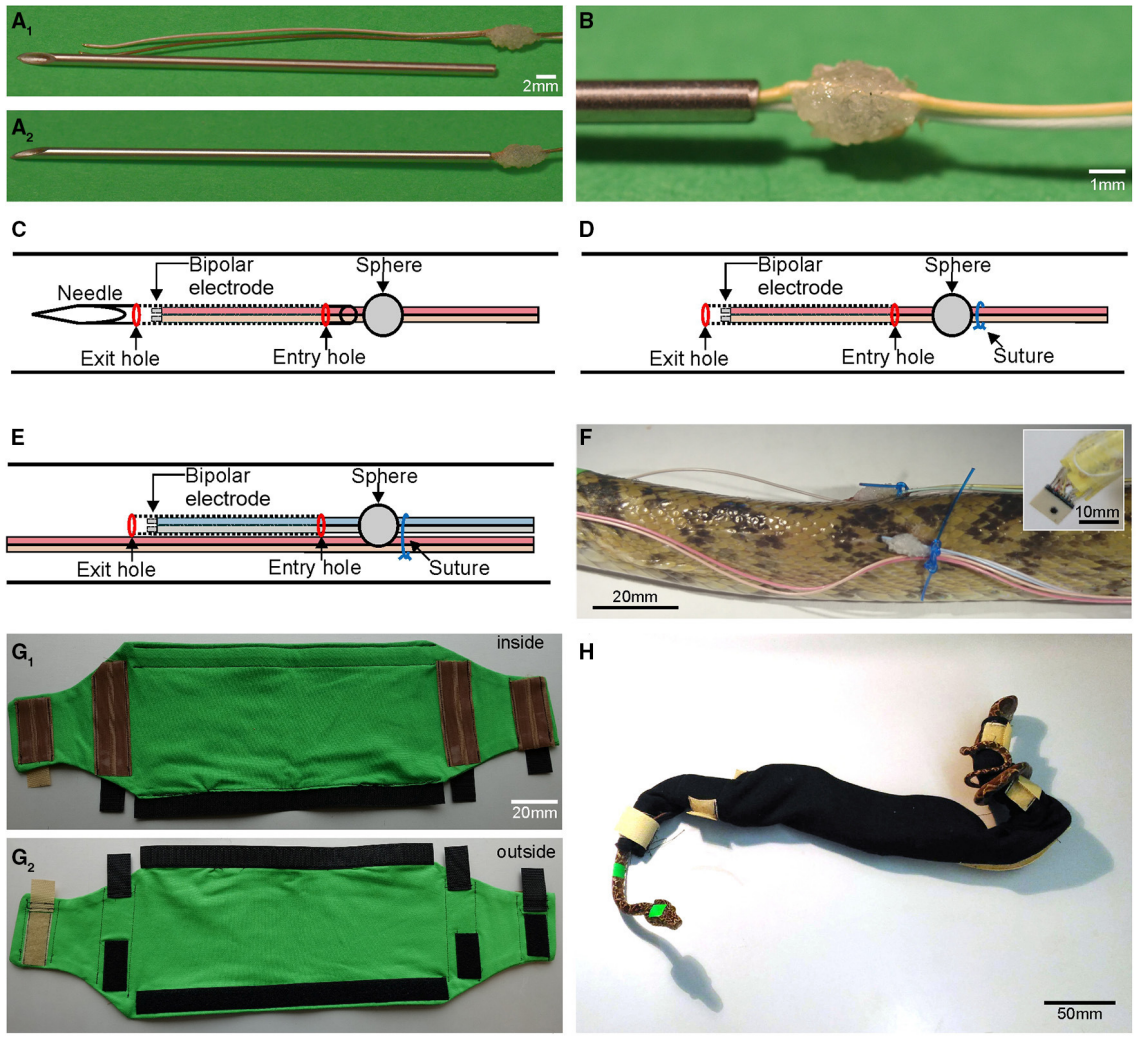
Custom-built items required for chronic mounting and anchorage of multiple EMG electrodes. **(A**_**1**_**)** Shortened injection needle and EMG electrodes with attached cyanoacrylate sphere that ties two cables to form a bipolar electrode. **(A**_**2**_**,B)** Needle with cables inserted up to the cyanoacrylate sphere **(A**_**2**_**)** and higher magnification of the cyanoacrylate sphere **(B)**; position of the sphere and needle length is adjusted such that the electrode tips remain inside the needle during the implantation process. **(C,D)** Schematic depicting the first implant position, with the needle, containing the bipolar electrode, located subdermally (dotted lines) between the entry and exit hole **(C)**; after removal of the needle, the bipolar electrode remains in the tunnel between the skin and epaxial muscles **(D)** invariably fixed in place by the suture (blue circle); schematic of the second, more caudal implant position **(E)** with cables of the first bipolar electrode pair (light red and beige) affixed to the skin with the suture that also holds the second implanted electrode pair in place. **(F)** Photograph of the second implant site in an Amazon tree boa depicting the wiring, cyanoacrylate sphere and suture as shown schematically in **(D,E)**; the inset depicts the connector at higher resolution. **(G**_**1**_**,G**_**2**_**)** Inside **(G**_**1**_**)** and outside view **(G**_**2**_**)** of the “snake sleeping bag” for Amazon tree boas used to prevent accidental removal of cables by the snake; brown silicone stripes increase friction and when closed prevent the snake from exiting the bag; black and beige hook and loop fasteners allow for easy and fast closing/opening of the bag. **(H)** Photograph of an Amazon tree boa inside the “snake sleeping bag”.

Electromyography electrodes and implantation needles were prepared 1 h before application of the anesthetic drugs to minimize the time required for the implantation process. For this procedure, thin flexible cables were used to avoid an impairment of the natural motility and to support the large number of implanted electrodes. The absence of any impact of the inserted recording electrodes on natural motion dynamics and pattern was verified by a qualitative estimation of the locomotion capacity before and after the implantation. For connecting the electrodes, Omnetics Neuro NanoStrip connectors (A79021-001, Omnetics, Minneapolis MN, USA) were used, which consisted of a miniaturized connector (7.4 × 4.4 × 1.8 mm), pre-wired with 18 cables [gold plated copper alloy, length: 46 cm; ø 0.160 mm; 0.3 mm including polytetrafluoroethylene (PTFE) coating]. Each cable was electrically shielded with an individually colored layer of biologically compatible PTFE coating and was thus protected from producing a short-circuit with other wires. The total weight of the wires including the connector was 2.8 g which equals 1.6–3.5% of the snakes' bodyweight and was thus easily supported even by the smallest snakes employed for the study. The cables were used in pairs, such that the cut ends formed a bipolar EMG electrode (Figures 1A–F). Using a connector with 18 cables generated eight bipolar electrodes with one of the two remaining electrodes used as ground wire, and the other as backup in case the ground electrode becomes damaged or removed during the recordings.

In order to secure the EMG electrodes in a close and constant relative position to each other, cables were affixed to each other using a small drop of cyanoacrylate glue (Figures 1A,B). To prevent the cables from undesired, accidental removal (discussed below) a solid spherical object was formed from additional drops of glue, whereupon the drying process was accelerated through application of small granules of sodium bicarbonate (Supplementary Video 1). The distance between the sphere and the tip of the EMG electrode determined the extent of the cable section that was subdermally implanted and served to ensure a consistent length of all cables within a given animal. In our experiments on both species of snakes, implanted cables with a length of 40 mm were employed, allowing a robust fixation without affecting natural movement capabilities.

The steps for producing an electrode were as follows: the position of the sphere on the cable was visually marked while aligning the two cables. At the same time a small drop of glue was applied to both cables at the marked position. Both cables' relative length was ensured to remain invariant relative to each other, as this would have caused problems with cable management during the experiments. After application, while the small drop of glue hung from both cables in a half-spherical form, a pinch of sodium bicarbonate was sprinkled from above onto the glue to facilitate solidification and drying. This caused the downwards hanging glue to be pulled upwards into a half-spherical form, due to the combination of surface tension and instantaneous fixation. Rotation of the cable and repetition of the procedure ensured a small but strong hold that was spherical in form (Figure 1B). Care was taken to avoid creating an excessively large sphere (>3 mm) since an oversized sphere causes a configuration where an undesired space between the snake's body and the cable might be formed. This would allow another object to insert itself between the cable and the snake and to generate sufficient force to remove the cable. A sphere diameter of ~2–3 mm was found to be most suitable for stable cable implantation. Any rough edges were removed from the sphere by careful sanding with high-grit sandpaper. The soldered connectors for the cables were oriented in relation to the snake such that the cable bundles were directed toward the caudal end of the snake to daisy-chain the implanted electrodes with the amplifier through a connecting cable approaching from the caudal part of the animal. This helped preventing an entanglement and minimized cable tension potentially caused by animal movements as the caudal part of the snakes generally showed less displacements in space during provoked movements compared to the rostral part of the body. To prevent bundles of excess cable to extend caudally, the length of the cables was trimmed according to the distance between the different electrode insertion points: if the electrodes were directed from rostral to caudal at a distance of 6 cm from each other with a bipolar electrode on the left and right side of the axial column, respectively, then cables 1–4 remained at a length of 46 cm, cables 5–8 were trimmed to 40 cm, and cables 9–12 were trimmed to 34 cm, etc. For the shorter rattlesnakes, the same cable lengths were used. Excess stretches of cable were fused together behind the caudal most implantation site and wrapped in parafilm. The insertion of the cables was performed with a hypodermic needle (Figure 1A). The diameter (outer diameter: 1.2 mm) of the needle was just wide enough to accommodate the two cables that formed a bipolar electrode pair. The plastic Luer-lock connector was cut off with a sanding disk attached to a rotary tool preventing a closure of the internal canal, such that the needle approximated a hollow sewing needle.

### Anesthesia

Prior to the electrode implantation, snakes were placed in an induction chamber and pre-anesthetized with 2 ml isoflurane (Isothesia, Henry Schein Vet, Hamburg, Germany). As soon as the tail-pinch reflex ceased, snakes were intubated with a cat catheter (diameter: 1.2 mm) connected to an isoflurane vaporizer (Isotec-3, Völker GmbH, Kaltenkirchen, Germany). The isoflurane concentration provided by the vaporizer was set to 2.0–2.5%, to ensure adequate surgical anesthesia throughout the entire duration of the implantation process. Additionally, Carprofen (Carprosol, cp-pharma, Burgdorf, Germany) was administered (2 mg/kg body weight, i.m.) for analgesic treatment 1 h before the start of the implantation and once thereafter every following day throughout the entire period of EMG recordings.

### Electrode Implantation

Before electrode implantation, all surgical tools, needles and wires were sterilized through submersion in a disinfectant (Perfektan TB, Dr. Schumacher, Malsfeld, Germany) for at least 5 min. For the electrode implantation, the sharp end of the needle was used to penetrate the skin of the snake at predetermined positions at the side of the body and was subdermally guided over a distance of 4.5 cm (Supplementary Video 2). Great care was taken to prevent undesired penetration of muscles. The needle was then pushed from caudal to rostral along the longitudinal axis of the snake's body. After insertion of the needle for 4.5 cm, the sharp end was pushed outwards again to exit the skin. Thereby the needle entered and exited the skin simultaneously at two points to form a subdermal tunnel. Using a stereo microscope, the bipolar electrodes were inserted through the cut end of the needle until the sphere was flush with the end of the needle (Figure 1C; Supplementary Video 2). At this point, the sharp end of the needle was grabbed with small surgical hemostats and was fully pulled through the rostral penetration site. The inserted cables remained underneath the skin as the sphere was unable to traverse through the caudal penetration entrance generated by the needle (Figure 1D). The electrodes were thus precisely positioned underneath the skin at a distance of 4 cm from the initial penetration site through the skin following the tunnel created by the needle. Overall, this process was performed 10 times to insert the bipolar electrodes (eight penetrations with two cables implanted at each penetration site) and the two single, separate ground electrodes (two penetrations with one cable implanted at each penetration site) resulting in a total of 16+2 (18) implanted cables (Figures 1E,F). In this proof-of-principle, study the electrodes were approximately placed on the surface of the *semispinalis-spinalis* (SSP) muscles. If necessary, precise electrode positions could be determined by dissection or X-ray imaging.

### Cable Fixation

In order to prevent cable removal, a suture (Daclon Nylon, Monofilament Non-absorbable, USP 2/0, SMI AG, St. Vith, Belgium) binding skin and cables was placed at the side of the glue sphere opposite to the cable's entry hole to tightly hold the two cables in place (Figures 1D–F). We used non-absorbable nylon monofilaments, because of its high tensile strength, manageability, and good tissue compatibility. The suture needle was inserted laterally from the center of the cyanoacrylate sphere such that it appears that the suture will bisect the sphere. Before the initial knot of the suture was tightened, the suture thread was positioned to the side of the sphere, such that the sphere was centered between the electrode penetration site and the suture loop (Figure 1D). The suture thereby applied a slight pressure against the sphere, which facilitated a tight arrest of the open end of the cable underneath the skin and minimized the likelihood of cable removal. Such an unfortunate circumstance would occur when an open space would form between the skin and the end of the cable separated by the sphere. Cables from rostrally implanted electrodes were bundled and affixed to the body (Figures 1E,F). Further intermediate sutures were placed at locations between adjacent bipolar electrodes to form bundles of cables from several target sites. Care was taken to create sufficient slack, such that the cables allowed sufficient mobility when the snake formed pronounced body loops. This was determined during anesthesia by laterally bending the body of the snake in both directions. At the end of the implantation process, extending cables proximally to the connectors were coiled and carefully wrapped in parafilm (Figure 1F, inset).

### Postsurgical Recovery

Following implantation, the isoflurane concentration was set to 0%, which caused complete recovery of the snakes from the anesthesia within a few minutes. During the experimental procedure each snake was regularly and systematically monitored. There was no alteration of the health of the snakes nor any obvious inflammatory signs at the site of the implanted electrodes. It was important that during the recovery period and prior to the experimental trials on successive days the snake's movements remained restricted to prevent the occurrence of forces onto the implanted cables. This measure assisted in preventing accidental removal of cables and consequential damage of the skin. In chronic experiments that lasted for several days, snakes were kept overnight in quarantine cages. A particular challenge during the overnight rest was the prevention of the cables from becoming entangled in cage enrichments, e.g., branches. A major advancement for the performance of chronic measurements across several days was therefore the reliable and faithful affixation of recording electrodes and cables at all times. This was particularly challenging at night in the home cage when the animals were essentially unobserved. Instrumental for successfully maintaining all electrodes in place and functionally intact was the use of an individually adjusted “snake sleeping bag,” which prevented impairment and deterioration of electrode placements and thus ensured the continuity of the recording condition over the experimental period of up to 4 days (Figures 1G,H). The maximum temporal extent for EMG recordings of 4 days was determined by the maximal duration that the legal body approved for these experiments and thus was not based on technical limitations of the described method.

The “sleeping bag” was made out of fabric and securely covered the segment of the body where the cables were inserted. The “sleeping bag” tightly kept the electrodes and cables in place but still allowed the animal to perform undisturbed locomotor movements. For arboreal species with a more elongated and slender body such as the Amazon tree boa (*Corallus hortulanus*) employed in our study, the use of such a bag was highly beneficial as these snakes generally use their prehensile tail to wrap around objects, as well as around their own body. The “sleeping bag” was constructed from a stretchy jersey fabric. Critical for the stable positioning of the bag was the extension of the snake out of the fastened shut bag on both sides (Figure 1H). If only the head were to come out of the bag, then the snake could still insert the highly flexible tail through loops that would form with the inserted cables, potentially removing the latter.

At the points where the head and tail exited the bag, hook and loop fasteners were attached to the outside of the bag (Figure 1G_2_). On the inside of the bag in relation to the hook and loop fasteners were silicone stripes (Figure 1G_1_). These stripes helped to hold the bag in place around the body such that the snake was unable to slither out. The middle section of the bag consisted of a large compartment that was closed by a long strip of hook and loop fasteners running parallel to the extent of the compartment. The size of the bag was adjusted to the size of the snake (0.9–1.4 m SVL) such that the animal was not able to fully extend its individual body segments. This prevented the snake from forming several S-shaped curves at body midsection and to pull the tail in through the bottom of the bag. As the diameter of the body directly behind the head of Amazon tree boas is smaller than that of the midsection, a well-fastened hook and loop closing mechanism prevented the animal from slithering out of the bag during forward movements. The diameter constriction of the body behind the head also prevented the snake from pulling the latter back into the closed containment. Besides ensuring a strong and uniform closing mechanism, hook and loop fasteners facilitated a quick and easy opening and closing of the bag to insert and remove the snakes. For terrestrial and more heavy bodied snake species such as Western diamondback rattlesnakes, also employed in this study, the use of a “sleeping bag” was not necessary. These snakes lack an extensive tail region that allows being wrapped around objects or the own body. During the post-surgical period, we noticed in fact that these snakes remained rather coiled-up overnight, thereby minimizing the risk of electrode impairment.

### Data Collection

**Figure 2:**
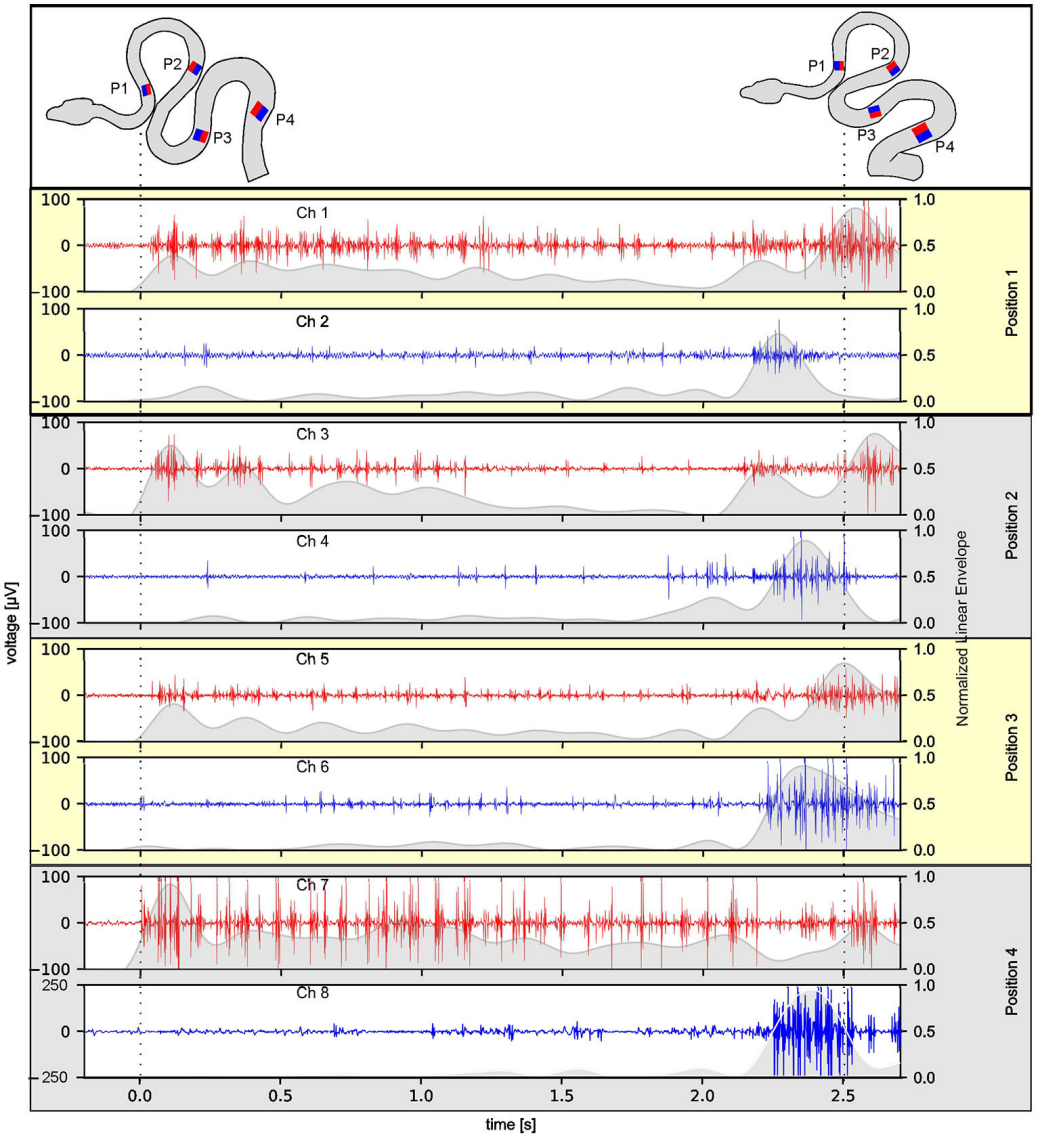
Reliability of multi-electrode EMG recordings during stimulus-provoked changes of the snake's (Amazon tree boa) body formation. Representative recordings at four rostro-caudal positions (P1–P4 in the top scheme) on the left (red) and right side (blue) of an individual snake; the scheme of the body formation before (top left) and during turntable movement (top right) was reconstructed from videos recorded simultaneously with the 8-channel EMG 3 days after electrode implantation; dashed lines indicate time steps of the video frames used for reconstruction of the body formation. The envelope of the recorded EMGs was normalized to the maximum value, per channel, respectively, and was plotted as gray overlay onto the raw data.

In the current pilot study, eight bipolar electrodes and two additional ground electrodes were implanted as described above to demonstrate the constant quality of EMG recordings over several days. The bipolar electrodes were inserted in a pairwise fashion on the left and right side of the axial column at a dorso-lateral position (Figure 1F). To identify each electrode pair, electrodes were referred to a particular “position,” as shown in Figure 2. The insertion points for the four rostro-caudal positions were determined by the following distances along the axial column starting from directly behind the skull (0 cm): 6, 17, 28, and 39 cm. Since the rattlesnakes used in this study were about half the length of the boas, the respective distances for the corresponding rattlesnake implantations were 5, 10, 15, and 20 cm. The two monopolar ground electrodes were inserted at ~45 cm in Amazon tree boas (~25 cm in rattlesnakes) behind the head on both sides of the body. After implantation, each snake was allowed to recover overnight, and EMG data were collected over a period of four consecutive days. Recorded EMG signals were amplified by a factor of 192 and digitized (20 kHz) by a 16-channel amplifier board with bipolar (differential) inputs (RHD2216, Intan Technologies, Los Angeles, California). Digitized EMG signals were forwarded to a USB-interface board (RHD2000, Intan Technologies, Los Angeles, California) and stored on computer using the USB interface board software provided by Intan Technologies. The recorded signals were processed by a 50 Hz notch filter followed by further processing with a fourth order Butterworth band-pass filter with a lower end of 20 Hz and upper end of 850 Hz. Recordings were down-sampled by the intan software to 4 KHz to ease plotting of the data, though the original data were stored for further analysis. To calculate the envelope of the recorded EMGs, the data was rectified and a fourth order Butterworth low-pass filter (8 Hz) was applied. Bias caused by rectification was removed by using the data before the start of the motion stimulus device (see below) to calculate a mean. This mean was then subtracted from the full data range. Subsequently, the values were normalized between 0 and 1 to facilitate comparison between electrodes and individuals.

A custom-built motion stimulus device (turntable) was used to provoke compensatory postural reactions driven by stereotypic muscle activity for the assessment of EMG quality. Accordingly, the snake was placed on a branch that was positioned in the vertical rotation center of the turntable. The turntable had a dimension of 30 × 30 cm and was driven by a brushless DC motor (Model:3268G024BX4 CS, Faulhaber, Schönaich, Germany). A MATLAB script (MATLAB ver. R2016a, Mathworks, Natick, Massachusetts, USA) was used to control turntable movements. The standard stimulus consisted of a sinusoidal rotation in the horizontal plane with a positional excursion of ±60° and a frequency of 0.1 Hz (period of 10 s). A spatially invariable infrared stimulus (IR-Emitter, Steady State IR Source, Model EK-5270, Laser Components GmbH, Olching, Germany) was presented in front of the snakes to attract the attention of this infrared-sensitive species. Since the snakes focused on the position of the IR-Emitter, the head remained relatively stable in space during turntable rotation. This was achieved by the snakes through an activation of compensatory movements of the head/body involving the most rostral body loops. Thus, turntable motion caused the snake to decrease or increase the diameter of its S-shaped body loops, which resulted in a relatively stereotyped motor behavior, ideally suited to assess the quality of the EMG recordings. To reconstruct the change in body shape, a video camera (Basler Ace acA1300-200uc, Basler AG, Ahrensburg, Germany) was mounted above the turntable and was temporally synchronized with the EMG recording device.

**Figure 3:**
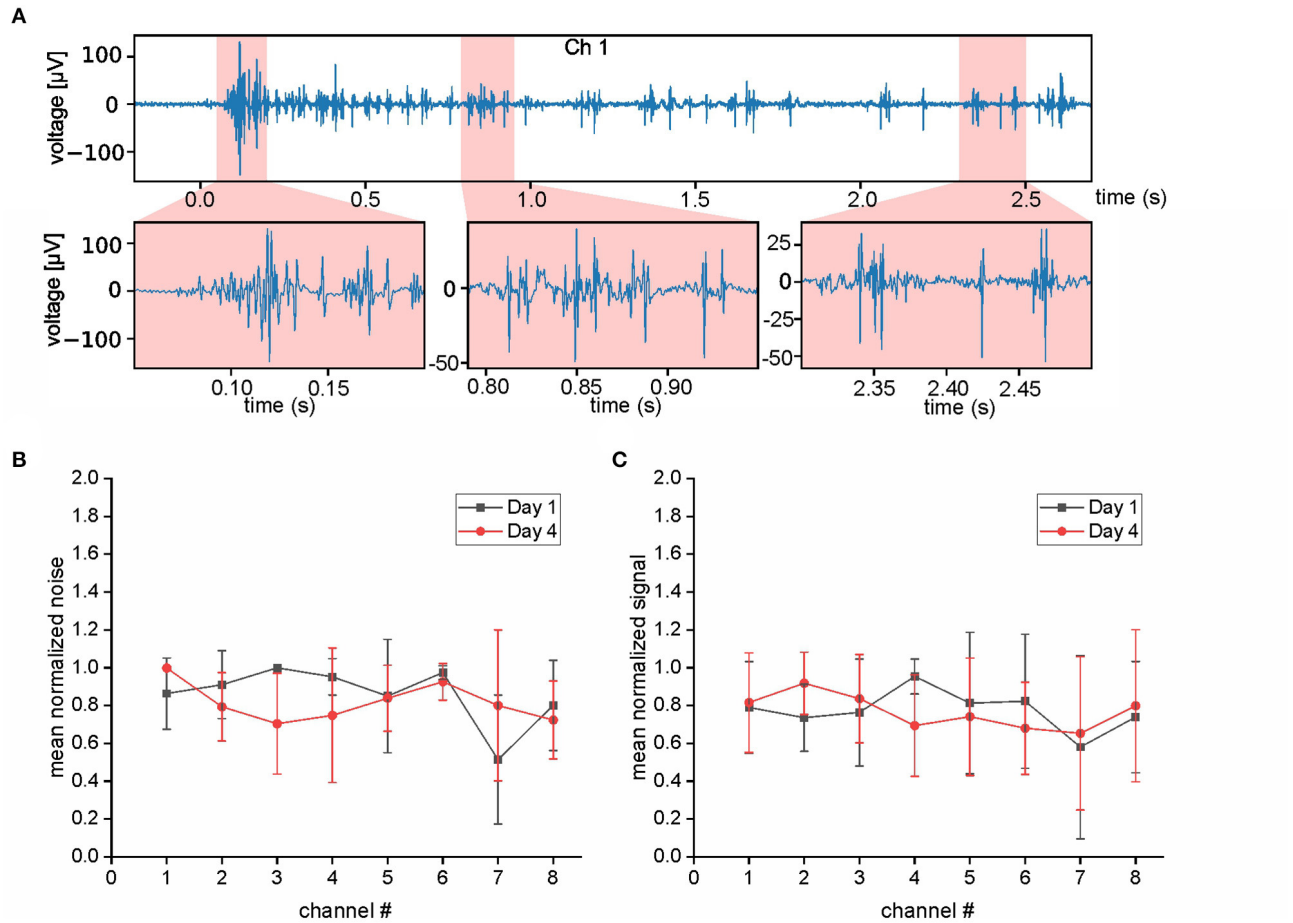
Evaluation of EMG quality. **(A)** Selected period of EMG activity (top trace) and at higher temporal resolution (bottom traces). **(B,C)** Evaluation of the electrode performance at day 1 (black) and day 4 (red) after implantation. Data that consisted mostly of noise **(B)** was analyzed separately from data with actual strong EMG activity **(C)**. The analyzed data was recorded from muscle activity during strikes of an Amazon tree boa; note the similarity of the values at day 1 and 4 for both parameters.

To verify that the described chronic recording technique is also applicable to other snake species with a different body shape and lifestyle, we performed EMG recordings in the Western diamondback rattlesnake—a heavier bodied species with a ground-based lifestyle. This species, however, turned out to be unsuitable for turntable experiments, because of a lack of robust postural stabilization as performed by the Amazon tree boa. We therefore designed a different task and recorded EMGs during aggressive strikes from both, Amazon tree boas and from Western diamondback rattlesnakes. This highly dynamic behavior was chosen, because it could be easily provoked in both species and due to the high kinematic profile posed a particular challenge for the stability of the implanted electrodes. In these experiments, the Amazon tree boas rested on the same branch as in the turntable experiments. To account for the ground-based lifestyle, Western diamondback rattlesnakes were placed directly on the flat surface of the stationary turntable. An infrared stimulus (IR-Emitter, Steady State IR Source, Model EK-5270, Laser Components GmbH, Olching, Germany) in front of the animals at a distance of 10–20 cm was used to attract their attention and to elicit aggressive targeted strikes. The video camera (see above), mounted above the turntable and synchronized with the EMG recordings, was used to capture the strikes for offline analysis. To determine when strike activity started in the EMG signal a measure of 1.5 standard deviations above the mean of the total signal capture was used. This metric alone has many false positive correlations. To mitigate these aspects, a sliding window of 35 ms was used. The start of EMG activity of the strike was only marked once all timesteps in the window were above threshold. Furthermore, the search for the strike start was started 250 ms prior to the time of visual strike start. The data set obtained during the strike behavior of Amazon Tress Boas (*N* = 4) was chosen for the evaluation of the electrode performance across successive recordings days. Accordingly, the data on the strikes recorded at the first day after electrode implantation was compared with those recorded 4 days after implantation. To differentiate data that consisted mostly of noise from data with actual strong EMG activity, the first second of each recording without stimulation or movement was selected and referred to as “noise” (Figure 3B). In comparison, the time period from the visual start of the strike until the time of maximal extension of the snake during a strike was referred as “signal” (Figure 3C). The mean amplitude of these time periods was calculated separately for each individual animal. The mean amplitude of the “noise” (respectively, “signal”) from day 1 was compared to the data obtained on day 4 by normalizing to the maximal value. Normalized data was then averaged across the four tested individuals.

At the end of both types of experiments over a period of 4 days, snakes were decapitated under deep isoflurane anesthesia (5%) using an animal decapitator (Small Animal Decapitator, Stoelting Co., Wood Dale, IL, USA).

## Results

**Figure 4:**
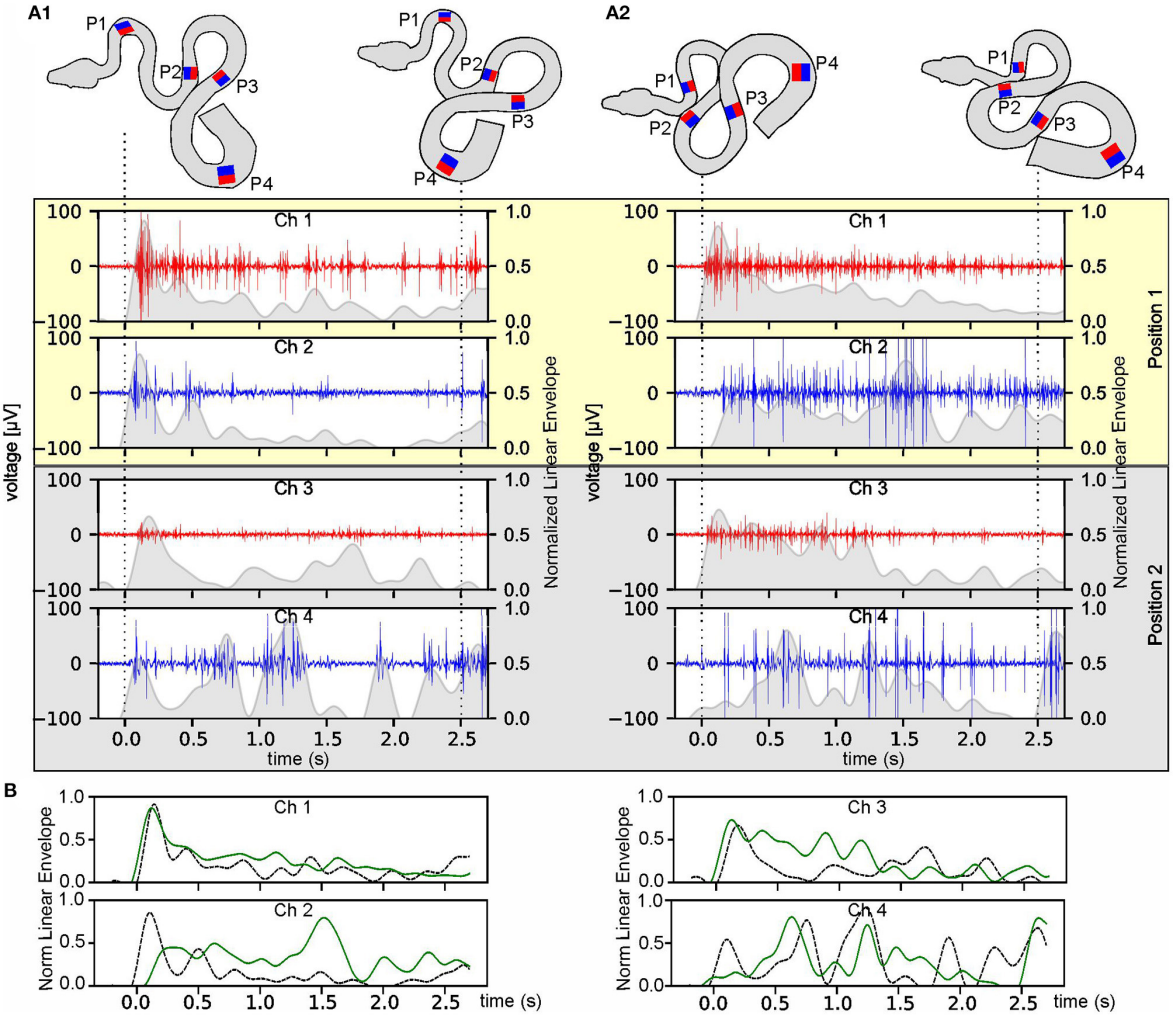
Temporal robustness of multi-electrode EMG recordings during stimulus-provoked changes of the snake's body formation. **(A)** Muscle activity of an Amazon tree boa other than the one from which the data were presented in Figure 2 at day 1 **(A**_**1**_**)** and day 4 after implantation **(A**_**2**_**)**; recordings derived from the two most rostral positions (P1 and P2 in the top scheme) on the left (red) and right side (blue); the scheme of the body formation before (top left) and during turntable movement (top right) was reconstructed from videos recorded simultaneously along with the eight-channel EMG; the recordings from P3 and P4 are illustrated in Supplementary Figure 1; dashed lines indicate time steps of the video frames used for reconstruction of the body formation. The envelope of the recorded EMGs was normalized to the maximum value, per channel, respectively, and was plotted as gray overlay onto the raw data. **(B)** Overlays of the normalized envelopes of each channel at position P1 (channel 1 and 2) and P2 (channel 3 and 4) at day one (dashed black lines) and day four (solid green lines).

The success of simultaneous EMG recordings from eight bipolar electrodes along the snake body was initially demonstrated by data sets of muscle activity recorded from two different Amazon tree boas 1 day after electrode implantation (Figures 2–4). When resting on the branch prior to horizontal turntable movements, the recorded EMG showed only weak activity of units with small amplitudes, which sometimes was difficult to discriminate from concurrent noise (Figures 2, 4A_1_). However, following initiation of the sinusoidal rotational movement, snakes actively changed their body formation (Figures 2, 4A_1_), accompanied by a patterned axial muscle activity with a high signal-to-noise ratio (Figure 3A). There was no substantial change in the signal quality during the entire experiments, even when the recording electrodes were confronted with high forces that occur during fast strikes of Amazon tree boas (Figure 3B) that cover long-distances. The EMG revealed a bilaterally side-specific pattern, which consisted of an asynchronous muscle activity on the left (Figures 2, 4A_1_, red traces) and right side (Figures 2, 4A_1_, blue traces) at any one of the four rostro-caudal recording positions. The side-specific activity pattern was accompanied by a phase-difference of the respective myogenic potentials. When different individuals were positioned in the center of the rotation axis, such that a comparable body form was assumed, the recorded activity was similarly patterned (Figures 2, 4A_1_,A_2_; Supplementary Figure 1).

Likewise, EMG data, collected from a given animal at two separate days (day 1 and day 4 after the implantation) were used to evaluate the temporal consistency of the recordings during the experimental period (Figures 4A,B; Supplementary Figures 1A,B). Recorded EMG signals from the same channels appeared to be qualitatively very similar on the 2 days (Figures 4A,B; Supplementary Figures 1A,B). During the separate recording sessions, the muscle activity exhibited recording-site-specific high signal-to-noise ratios with variations across channels, nevertheless allowing a detailed pattern analysis by calculation of the envelope of the recorded EMGs. The variations in the activity pattern between day 1 and day 4 were larger at the caudal recording sites (P3 and P4, Supplementary Figure 1) as compared to the more rostral recording positions (P1 and P2, Figure 4) and were likely related to the more variable anchoring positions of the snake at the latter body positions.

**Figure 5:**
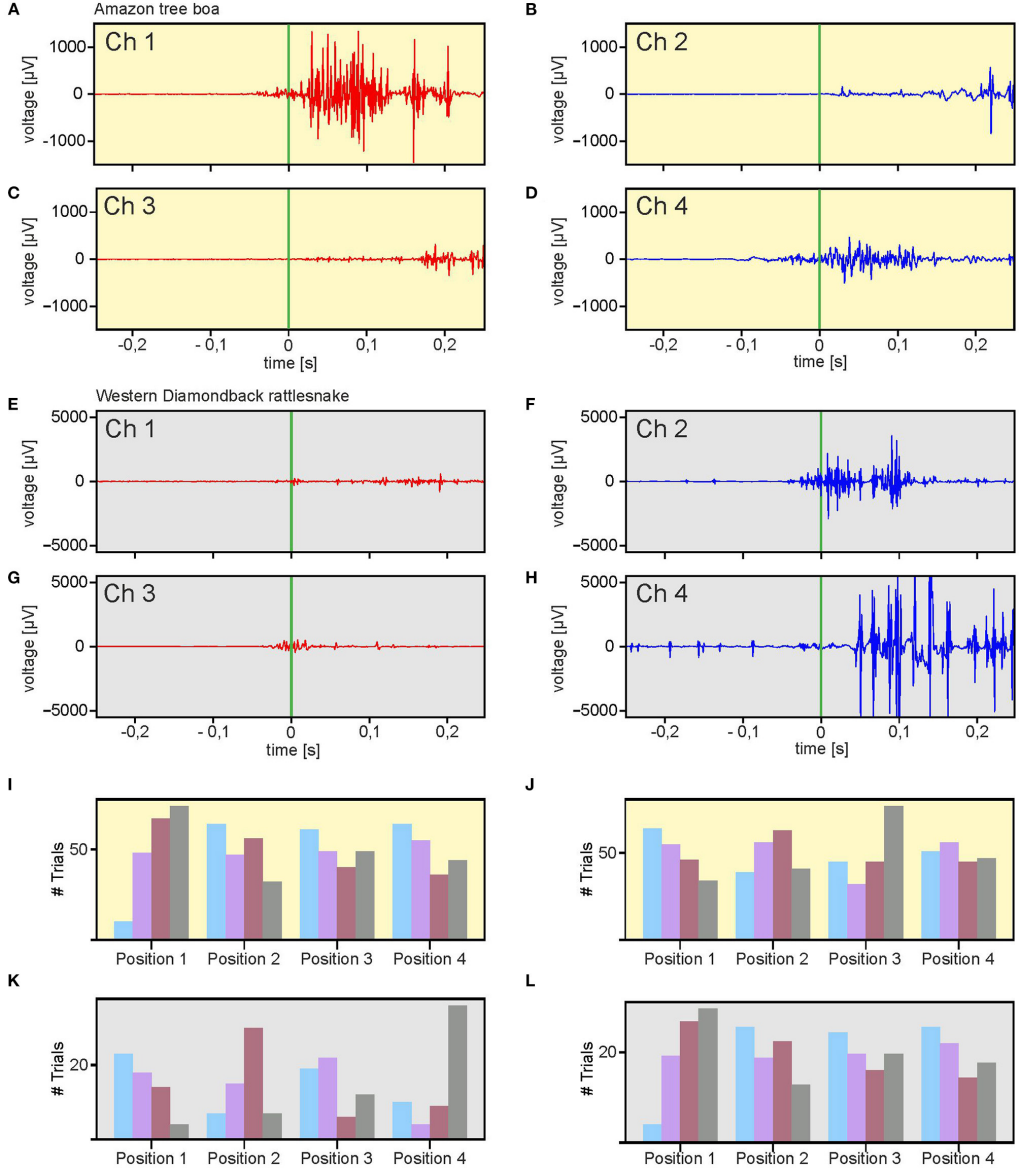
Multi-electrode EMG recordings during stimulus-provoked aggressive strikes. **(A–H)** Representative recordings from an Amazon tree boa at two rostro-caudal positions [**(A,B)**: Position 1; **(C,D)**: Position 2]. **(E–H)** Similar recordings as in **(A–D)**, from a Western diamondback rattlesnake. **(I–L)** Distribution of spatio-temporal muscle activation patterns along the body axis of all recorded strikes from the Amazon tree boa **(I,J)** and the Western diamondback rattlesnake **(K,L)**. The left and right sides of the body was classified for each recording position as fast-activation side **(I,K)** or slow-activation side **(J,L)** based on the temporal sequence of the activation. The order of activation is depicted by colored bars (blue = first, purple = second, red = third, gray = fourth). Green vertical bar in **(A–H)** indicates start of the snake strikes.

The data sets, obtained from simultaneous EMG recordings of eight bipolar electrodes during snake strikes (Supplementary Video 3) showed that the employed technique is sufficiently stable to also capture the muscle activity during highly dynamic movements (Figures 5A–H; Supplementary Figure 2) in a comparative approach in different snake species. In total, 199 strikes of the Amazon tree boa (*N* = 5) and 71 strikes of the Western diamondback rattlesnake (*N* = 2) were recorded. In contrast to the slow compensatory postural reactions provoked by a turntable rotation, the EMG activity during snake strikes was considerably shorter with the maximum activity at the time when the actual strike was launched (Figures 5A,D,F,G). Recorded EMGs did not differ across species and showed a strong activation with high amplitude EMGs at strike start. However, temporal activation of epaxial muscles did not occur simultaneously when the recordings of the left and right side at a particular positional configuration were compared. In the example shown in Figures 5A,B, high amplitude EMGs at strike start were only recorded on the left side of the snake (Figure 5A), The activation of the right side (Figure 5B) was considerably weaker with a delayed appearance of EMG activity at higher amplitude. This activation pattern was neither species- nor side-specific and appeared to be rather related to the specific loop-formation of the snake body. If the loop was formed in a mirror-image fashion, as the illustrated example of a rattlesnake strike in Figures 5E,F, the temporal activation sequence was inversed.

For further analysis, the recording positions on both sides of the snake were classified as fast-activation side or slow-activation side. The side (left or right) at which for a given recording position, EMG activity was encountered earlier than the corresponding activity on the other side was signified as fast activation side (Figures 5A,D,F,G) and the other side accordingly as slow activation side (Figures 5B,C,E,H). This classification scheme simplified the analysis and comparison of the spatio-temporal activation of strikes by both the Amazon tree boa (Figures 5L,J) and the Western diamondback rattlesnake (Figures 5K,L). Differently colored bars indicate the order in which EMG activity was recorded. Accordingly, e.g., for recording position 1 the blue bar indicates the number of strikes in which the first EMG activity occurred at this position. The purple bar, likewise, represents the sum of all strikes in which the EMG activity was recorded subsequently and so on. Since at each position bipolar electrodes were implanted on both sides of the snake, the differentiation between fast-side activation and slow-side activation was taken into account. The summation of the data obtained from all strikes of an Amazon tree boa (Figures 5L,J) and the Western diamondback rattlesnake (Figures 5K,L) demonstrated a high variability without a clear temporal activity pattern. Thus, we found no clear pattern that could be related to a wave-like activation either from rostral to caudal or *vice versa*. This would be the case when the firing order is strongly correlated with electrode position. Since such a correlation is absent in the distribution of fast-side and slow-side activation positions, there is no fixed sequential activation of consecutive body positions.

However, a noticeable difference emerged when the distribution of fast-side activation and slow-side activation was compared between the strikes of the Amazon tree boa and the Western diamondback rattlesnake. In the data obtained from the Amazon tree boa, there was hardly any correlation between the fast-side activation and the slow-side activation (Figures 5I,J). In contrast, a clear correlation was present in the data obtained from the strikes of the Western diamondback rattlesnake (Figures 5K,L). This was most evident, when looking at recording position 1 and 4 or the colored bars for the first (blue) and fourth (gray) electrode position. Thus, in the Western diamondback rattlesnake, the slow-side activation was spatio-temporally more linked to the fast-side activation than in the Amazon tree boa (see discussion below).

## Discussion

Following stable subdermal implantation of up to eight bipolar electrodes directly above epaxial target muscles, robust EMG activity with high signal-to-noise ratio was reliably recorded from all sites. The overnight coverage of the snake body ensured a position-invariant arrangement of all electrodes throughout the recording period, without loss or deterioration of the signals. The recording of comparable EMG waveforms across consecutive daily sessions confirmed the suitability of multiple subdermally implanted electrodes for the chronic acquisition of large sets of myogenic activity during natural motor behaviors in snakes. The robust recording of EMGs in rattlesnakes demonstrated the suitability of this method also for snakes with a different body shape.

Electromyography recordings from different individuals with comparable body formation and curvatures revealed similar activity patterns in response to turntable stimulation, suggesting 1) the presence of comparable task-dependent muscle activation patterns and/or 2) the recording of similar groups of muscle fibers. This indicates that the established method represents a suitable technique to reliably record and compare the contraction dynamics and temporal pattern of multiple equivalent groups of axial muscles across individual snakes. However, snakes assumed slightly different body configurations, when placed on the positioning branch during repetitive recording sessions. Thus, the recorded EMGs naturally showed variations across individuals and subsequent days of data collection related to the specific body loop formation.

The different body loop formation is also relevant for the interpretation of the fast aggressive strike of snakes. Since in our EMG data no clear fixed sequential activation of consecutive body positions became apparent, it is unlikely that the snake strike is activated by a fixed motor pattern. This would require that the snakes repetitively strike from identical positions with generalized loop formations. However, from our experience, especially with rattlesnakes, strikes can be initiated from any position independent from the actual loop formation. Thus, we hypothesize that the activation order is rather related to the distance that each loop adds to the strike, with larger loops being activated before smaller ones. This might also explain the difference, between fast-side and slow-side activation in both the Amazon tree boa and the Western diamondback rattlesnake. The comparison of the coordination of the slow- and fast-sides of the strikes provides information about how the first (fast-side) activity is being counteracted by the delayed (slow-side) activity of the other side. First activation of the outside of a loop pushes the snake into a more linearly aligned position. If that would not be counteracted by an opposing force, the snake would be unable to maintain this linear position and this particular section of the body would go past the midline to form a new loop at the opposite side of the body. Thus, the delay of the slow-side activation would depend on the actual loop size. This indicates that it takes more time for the body to straighten a big loop in comparison to a small loop. Therefore, counteraction by slow-side activity would be delayed when a big loop has to be straightened during a strike. Thus, the difference between the Amazon tree boa and the Western diamondback rattlesnake might be explained by the loop size before launching a strike. Smaller loops cause a slow-side activation with a shorter delay and therefore result in a stronger correlation between fast-side and slow-side activation. However, a more detailed study design that includes high-speed video acquisition from multiple cameras for a detailed analysis of loop formation would be necessary to confirm this hypothesis considering the actual strike model (gate vs. tractor tread) as described by Kardong and Bels (1998). This would also allow for a comparative investigation of snake motor patterns with other neuromechanical models such as the undulatory sand-swimming of sandfish lizards, that has been analyzed in greater detail (e.g., Ding et al., 2013; Sharpe et al., 2013).

Nonetheless, independent of all activity details, the overall robustness of the recordings suggests that chronic compound EMG recordings of axial muscles for at least up to 4 days in snakes are possible and allow reliable acquisition of myogenic potentials of similar sets of muscle fibers at particular body positions in both employed species of snakes. It must be pointed out, however, that recordings of sEMGs, either using surface electrodes (e.g., in mammals; Staudenmann et al., 2010) or by placing electrodes directly onto the surface of the muscle (Biedermann et al., 1999, 2000; Scholle et al., 2001) as used here, will not yield sufficiently precise data to allow linking the recorded compound activity with individual muscle fiber bundles. Such an alignment clearly requires the implantation of electrodes into surgically exposed and thereby identified muscles, however, with likely detrimental consequences for motion patterns and trajectories. If locally restricted recordings from specific muscles or even from deep muscle tissue is required, classic hook electrodes should be preferentially employed (Loeb and Gans, 1986). However, implanting electrodes deep into muscular tissue impairs the mobility of the animals and thus the undisturbed execution of natural motor repertoires. Therefore, the specifically used EMG acquisition technique is generally a trade-off between muscle specificity and interference-free motor performance. Thus, our approach for electrode insertion, cable fixation, and potential snake movement restriction is therefore an elegant compromise that allows a considerable extension of the experimental period without undesirable loss of recording electrodes. This proof-of-concept study thus successfully demonstrated the possibility to faithfully record high-quality EMG signals over the course of several days at multiple sites along the body with a comparable suitability in two species of snakes with different body structures and life styles. Moreover, the occasional presence of distinctive large-amplitude single-units in the EMG recordings even allows sporadic identification of individual muscle fibers and thus the potential detection of task-specific differential contributions of muscle fibers with specific dynamic properties.

## Conclusion

The establishment of an improved multi-electrode implantation technique to record the EMG of snake axial muscles was highly successful and proved to be excellently suited for collecting high-quality muscle activity data for several days. Most instrumental for chronic recordings were the durable subdermal insertion of the recording electrodes as well as the use of the “snake sleeping bag” that efficiently prevented the loss of wires and ensured safe resting of the animal overnight. The latter invention also kept the cables unimpaired while the animal recovered from anesthesia and allowed multiple recording sessions over several days with the same configuration, likely recording the same muscle fiber bundles. In addition, the method caused no visible detriments of the health, vitality or mobility of the animal over an extended recording period. Thus, larger data sets can be recorded, which potentially reduces the number of experimental animals used in future studies. While this technique has less accuracy for the determination of muscle specificity in comparison to intramuscular EMG, it is more than sufficient to provide vital information on muscle activity that can be used to investigate muscle coordination in combination with concurrent high-speed video recordings of snake locomotion or strike movements.

## Data Availability Statement

The raw data supporting the conclusions of this article will be made available by the authors, without undue reservation.

## Ethics Statement

The animal study was reviewed and approved by the Regierung von Oberbayern (Gz.: ROB-55.2-2532.Vet_02-19-115).

## Author Contributions

GJ, HS, PS, and TK: conceptualization. GJ and TK: methodology, investigation, writing—original draft, and visualization. GJ: validation and data curation. GJ, HS, HL, PS, and TK: formal analysis and writing—review editing. PS, TK, and HL: resources. HS, HL, PS, and TK: supervision. TK: project administration. HS, PS, and TK: funding acquisition. All authors contributed to the article and approved the submitted version.

## Funding

This research was funded by a grant from the Bernstein Center for Computational Neuroscience (BCCN) Munich (Project B-T7) to PS, TK, and HS as well as by additional intramural funding from the Biomimetics Center at TUM to TK.

## Conflict of Interest

The authors declare that the research was conducted in the absence of any commercial or financial relationships that could be construed as a potential conflict of interest.

## Publisher's Note

All claims expressed in this article are solely those of the authors and do not necessarily represent those of their affiliated organizations, or those of the publisher, the editors and the reviewers. Any product that may be evaluated in this article, or claim that may be made by its manufacturer, is not guaranteed or endorsed by the publisher.
