# Supplementary material for: Chronic Multi-Electrode Electromyography in Snakes
Source: Front Behav Neurosci. 2022 Jan 7;15:761891. doi: 10.3389/fnbeh.2021.761891 (PMC8777293; doi:10.3389/fnbeh.2021.761891)
Supplement: Supplementary file 4 [file Data_Sheet_1.docx]

**Chronic Multi-Electrode Electromyography in Snakes**

Running title: EMG Snakes

**Supplemental Material:**

**Supplemental Figure 1** Temporal robustness of multi-electrode EMG-recordings during stimulus-provoked changes of the snake´s body formation. (a) Muscle activity of the same snake from which the data in Fig. 3 were obtained at day one (a_1_) and day four after implantation (a_2_); recordings derived from the two most caudal positions (P3 and P4 in the top scheme) on the left (red) and right side (blue); the scheme of the body formation before (top left) and after initiation of the turntable movement (top right) was reconstructed from videos recorded with the 8-channel EMG; dashed lines indicate time steps of the video frames used for reconstruction of the body formation. The envelope of the recorded EMGs was normalized to the maximum value, per channel, respectively, and was plotted as grey overlay onto the raw data. (b) Overlay of the normalized envelopes of each channel at position P3 (channel 5 and 6) and P4 (channel 7 and 8) at day one (dashed black lines) and day four (solid green lines).

**Supplemental Figure 2** Multi-electrode EMG recordings during stimulus-provoked aggressive strikes. (a-h) from the same individuals as presented in Fig. 4a-h). Representative recordings from an Amazon tree boa at two rostro-caudal positions (a,b: Position 3; c,d: Position 4). (e-h) Similar recordings as in a-d, from a Western diamondback rattlesnake. Green vertical bar in a-h indicates start of the snake strikes.

**Movie 1** Demonstration of cable preparation prior to surgical implantation. Paired wires that form a bipolar EMG electrode were attached to each other using a small drop of cyanoacrylate glue. To form a spherical object and to speed up the drying process, small granules of sodium bicarbonate were sprinkled from above onto the glue.

**Movie 2** Demonstration of surgical electrode implantation at a lateral position of the snake body. A sharp needle was subdermally guided over a distance of 4.5 cm to form a subdermal tunnel. Subsequently, a bipolar electrode was inserted into the tunnel until the sphere was flush with the end of the needle.

**Movie 3** Strike of an Amazon Tree Boa (*Corallus hortulanus*) during EMG recordings from eight bipolar electrodes.
