# Supplementary figures and images for: Chronic Multi-Electrode Electromyography in Snakes
Source: Front Behav Neurosci. 2022 Jan 7;15:761891. doi: 10.3389/fnbeh.2021.761891 (PMC8777293; doi:10.3389/fnbeh.2021.761891)

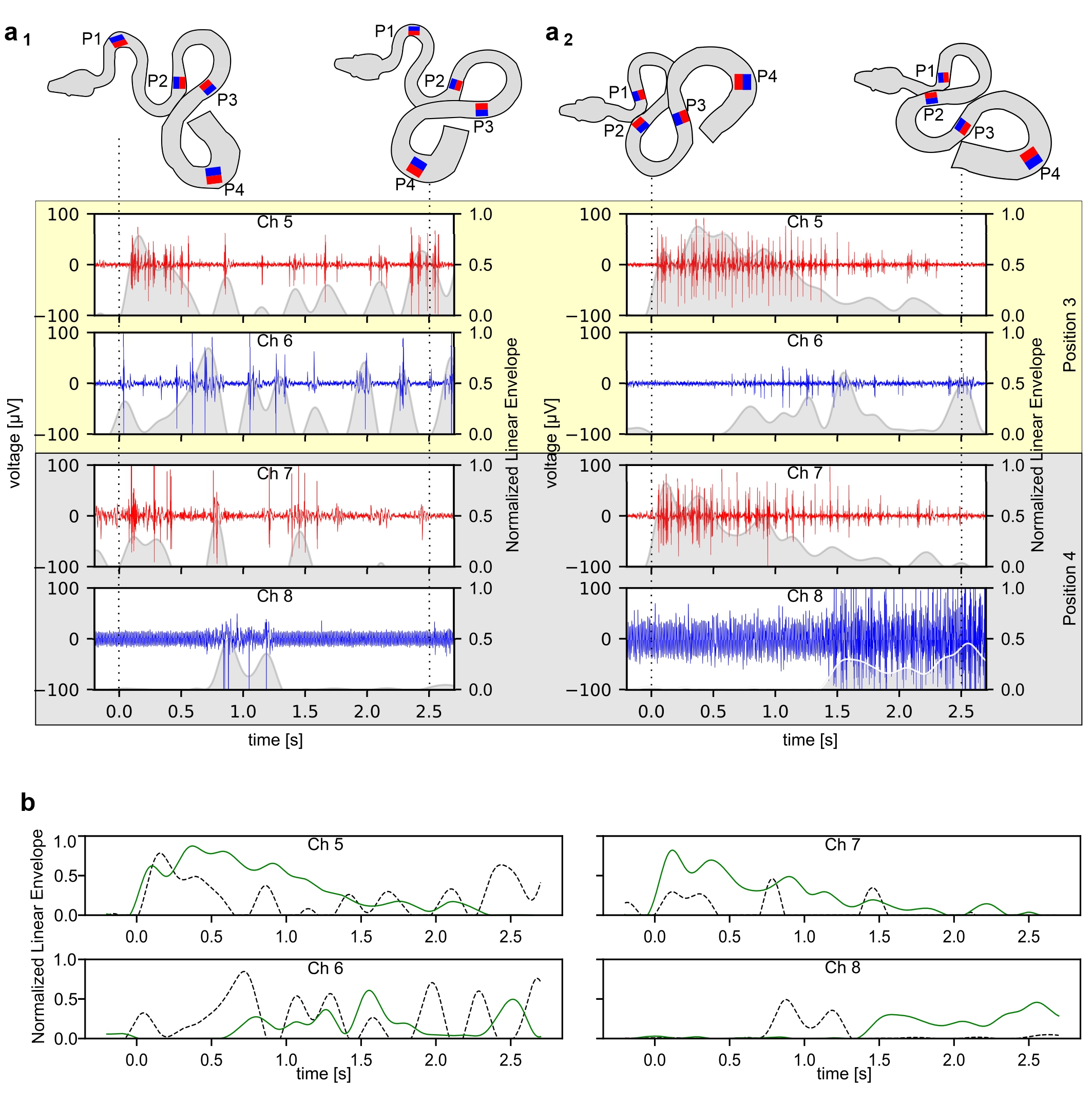

Supplement: Supplementary file 5 [file Image_1.JPEG]

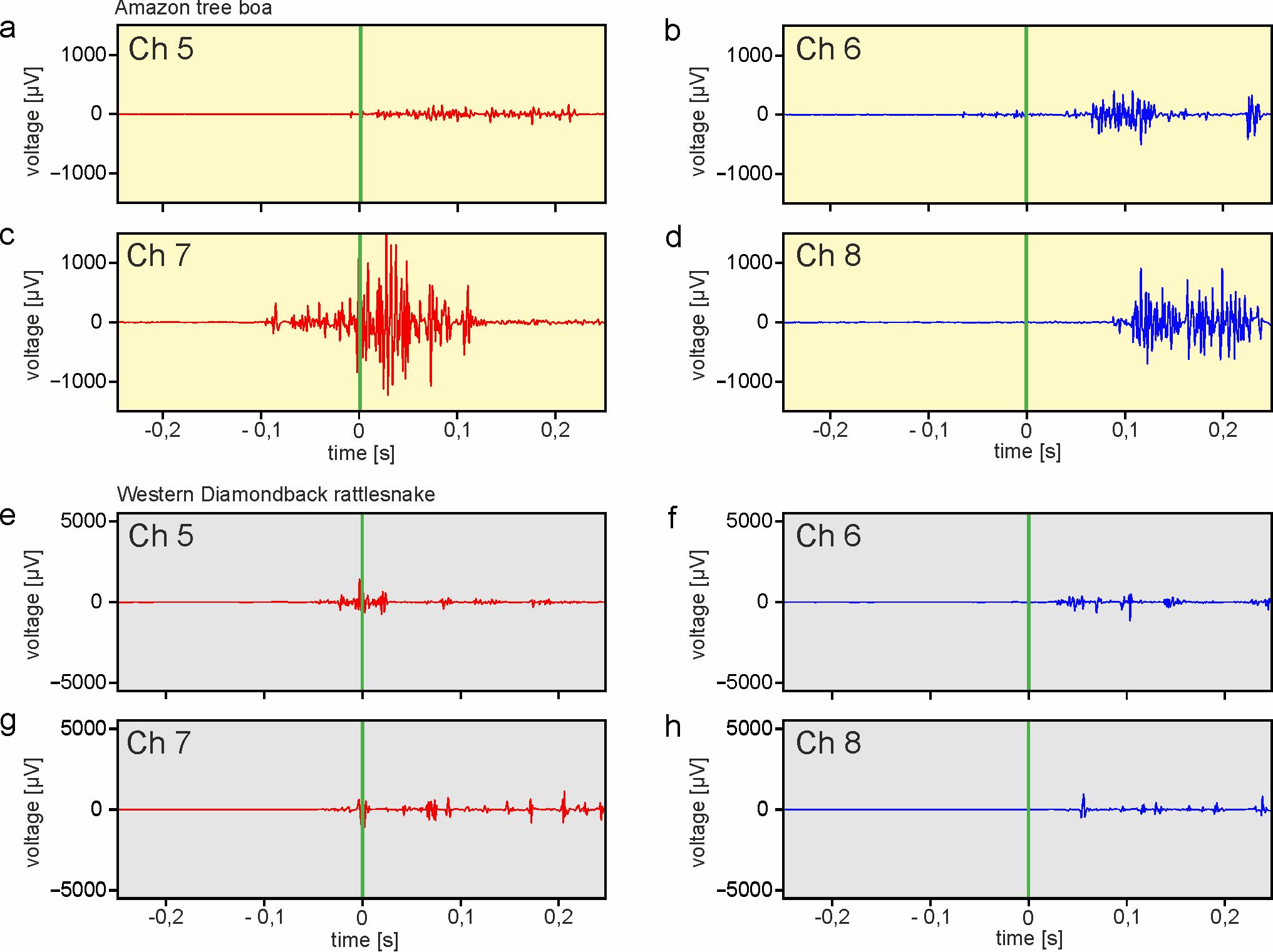

Supplement: Supplementary file 6 [file Image_2.JPEG]
